# Chelator Optimization and Therapeutic Potential of ^188^Re-FAPI for FAP-Targeted Radionuclide Therapy

**DOI:** 10.1021/acsmedchemlett.5c00486

**Published:** 2025-09-03

**Authors:** Steven H. Liang

**Affiliations:** Department of Radiology and Imaging Sciences, 1371 Emory University, 1364 Clifton Road, Atlanta, Georgia 30322, United States

**Keywords:** Fibroblast activation protein (FAP), FAP-inhibitor (FAPI), targeted radionuclide therapy, Rhenium-188

## Abstract

Fibroblast activation protein (FAP) has emerged as a highly promising molecular target for cancer theranostics, with current research prioritizing the optimization of FAP-targeted radiopharmaceutical pharmacokinetics. The development of diverse FAP inhibitor (FAPI) probes conjugated with therapeutic radionuclides has significantly advanced the field of FAP-targeted radionuclide therapy (FAP-TRT). Among available radionuclides, rhenium-188 has emerged as a particularly valuable theranostic radionuclide, offering the rare combination of economical availability, therapeutic β^–^ emissions (*E*_max_ = 2.12 MeV), and γ emissions suitable for SPECT imaging (155 keV, 15% abundance). The strategic development of ^188^Re-labeled FAPI compounds represents a promising approach to enhance the efficacy and clinical translation of FAP-targeted radionuclide therapy. A recent study has developed and evaluated four novel ^188^Re-labeled FAP inhibitors through rational structure optimization, which provided a cost-effective viable alternative to established therapeutic radionuclides in clinical oncology.

## Graphical abstract

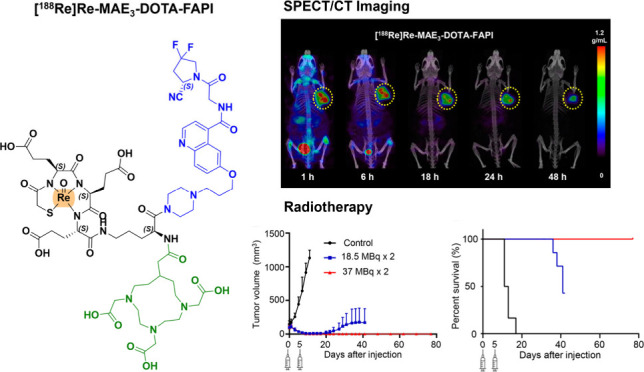

Fibroblast activation protein (FAP) is a type II transmembrane serine protease that is highly expressed in cancer-associated fibroblasts (CAFs), making it a compelling target for tumor stroma-directed therapy.^1,2^ FAP is overexpressed in over 90% of malignant epithelial tumors, whereas it shows negligible expression in normal tissues.^3^ Since 2018, the development of radiolabeled quinoline-based FAP inhibitors (FAPIs) with exceptional selectivity and high target affinity for FAP marked a significant advancement in nuclear medicine, offering novel diagnostic and therapeutic strategies for FAP-positive tumors.^4,5^ Represented by [^68^Ga]­Ga-FAPI-04, FAPI-based radiotracers exhibit both fast intratumoral accumulation and rapid normal tissue clearance, highlighting their potential as ideal theranostic agents. For successful therapeutic translation of FAPI probes, two critical barriers must be addressed: (1) to improve pharmacokinetic characteristics of the radioligand (2) to match with appropriate therapeutic radionuclides. Various strategies, such as modifying the parent structure, albumin-hitchhiking, dimerization, and covalent conjugation, have been developed to prolong the retention of FAPI tracer at tumor sites.^6–10^ The commonly used radionuclides for targeted radionuclide therapy (TRT) include yttrium-90, lutetium-177, and actinium-225. The clinical approvals of [^177^Lu]­Lu-DOTA-TATE and [^177^Lu]­Lu-PSMA-617 have propelled lutetium-177 to the forefront of therapeutic radiopharmaceutical development.^11,12^ However, the need for multiple treatment cycles (typically 4–8 per course) combined with the high cost of ^177^Lu therapies poses unsustainable financial burdens, limiting patient access and burdening healthcare systems. Therefore, the development of alternative, cost-effective TRT is imperative to improve treatment accessibility and provide clinically viable options.^13,14^

Rhenium-188 (Re-188) is a highly promising radionuclide for TRT due to its favorable nuclear characteristics and convenient production pathway.^15^ Rhenium-188 exhibits favorable characteristics for therapeutic radionuclide applications, with its physical decay properties (*t*_1/2_ = 16.9 h) combining high-energy β^–^ emissions (Eβ^–^max = 2.12 MeV) for effective tumor irradiation and concomitant γ-radiation (155 keV, 15% abundance) that enables simultaneous therapeutic monitoring through single photon emission computed tomography (SPECT) imaging.^16^ Re-188 can be conveniently obtained through a tungsten-188/rhenium-188 (W-188/Re-188) generator system utilizing an alumina-based column, which facilitates its widespread clinical availability. Rhenium shares similar chemical characteristics with technetium, as both belong to Group 7 (VIIB) of the transition metal series in the periodic table. This chemical similarity has led to the strategic pairing of rhenium-188 and technetium-99m as theranostic radionuclides.^17^ Several ^99m^Tc-labeled FAPI probes have been developed with the expectation that they could be readily adapted for therapeutic applications using rhenium-188.^18–20^ While ^188^Re appears chemically compatible with ^99m^Tc-based radiopharmaceuticals, practical implementation faces obstacles since the reduction of ReO_4_^–^ to reactive species requires substantially stronger reducing conditions than its technetium counterpart.^21–23^ To solve this problem, researchers have concentrated on creating novel chelators with improved chelating properties. N_*x*_S_4–*x*_ tetradentate donor type was often employed as chelation group to stabilize the oxorhenium­(V) core ([Re=O]^3+^). Mercaptoacetyltriglycin (MAG_3_) was used to label radiorhenium but with lower labeling yield. The PSMA-targeted probe ([^188^Re]­Re-PSMA-GCK01) confirmed that the labeling yield could be improved by serine substitution.^24^ Building on this, recent work has introduced alternative chelators such as MAS_3_ (serine backbone) and MAE_3_ (glutamic acid backbone), both designed to enhance the stability of [Re=O]^3+^ complexes and support the development of novel ^188^Re-labeled FAPI tracers (Figure 1).^25^

**Figure 1:**
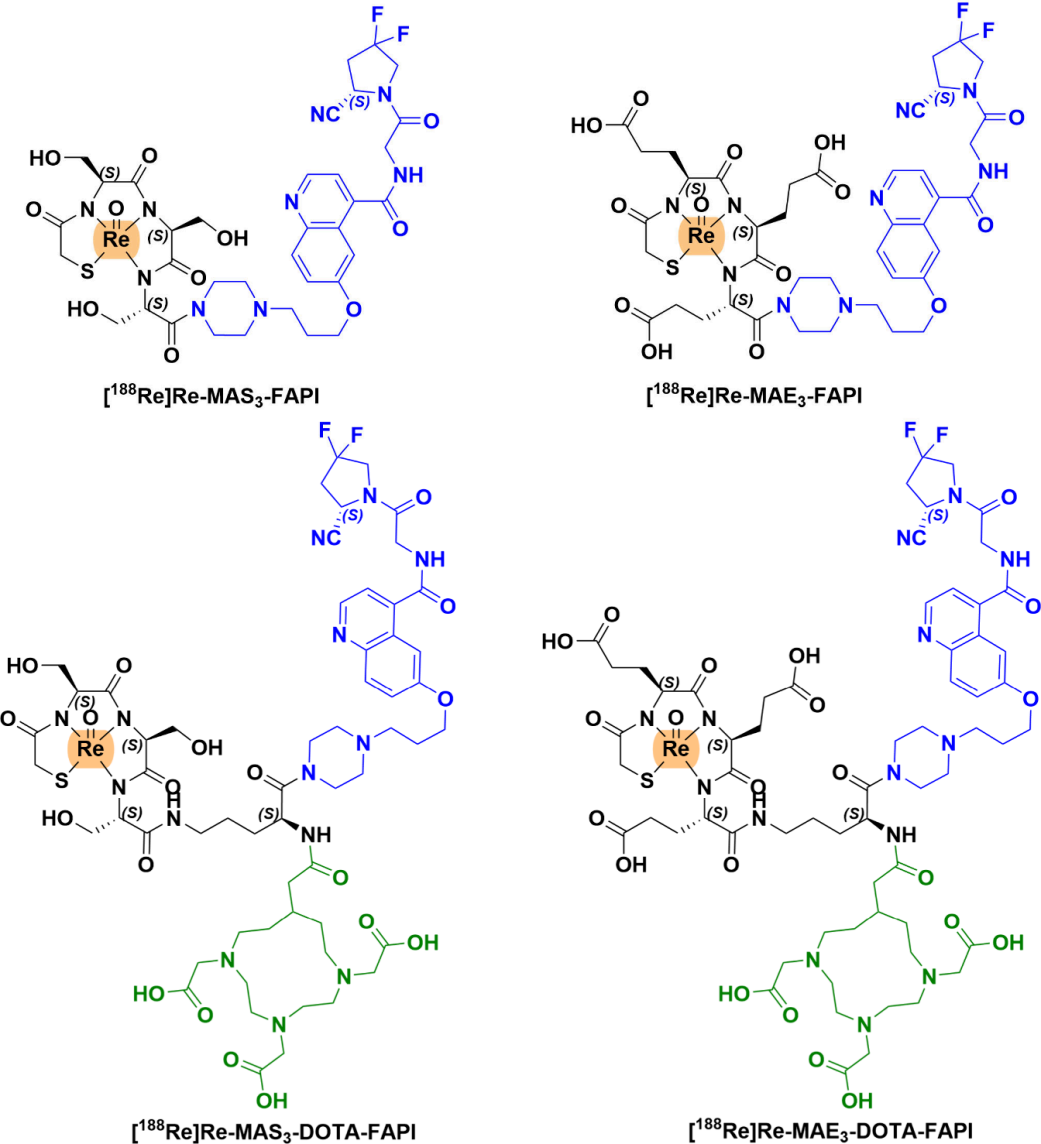
Chemical structure of ^188^Re-labeled FAPI tracers: [^188^Re]­Re-MAS_3_-FAPI, [^188^Re]­Re-MAS_3_-DOTA-FAPI, [^188^Re]­Re-MAE_3_-FAPI, and [^188^Re]­Re-MAE_3_-DOTA-FAPI). The figure was adapted from ref 25. Copyright 2025 American Chemical Society.

Based on the structure of FAPI-04, four ^188^Re-labeled FAPI derivatives were developed using MAS_3_ or MAE_3_ as chelators, with DOTA incorporated in two constructs to modulate pharmacokinetic properties (Figure 1). The corresponding [^188^Re]­Re-labeled complexes were successfully synthesized under optimized conditions (pH 2.0–3.5, 96 °C, SnCl_2_ as the reducing agent, 1 h), achieving high radiochemical yields (RCY > 80%). After purified by Sep-Pak Plus Light C18 column, the radiochemical purity (RCP) of [^188^Re]­Re-MAS_3_-FAPI was 92%, [^188^Re]­Re-MAE_3_-FAPI was 98%, [^188^Re]­ReMAS_3_-DOTA-FAPI was 91%, and [^188^Re]­Re-MAE_3_-DOTA-FAPI was 98%. Short-term stability (6 h) exceeded 85% for MAS_3_-based tracers and 90% for MAE_3_-based tracers, with [^188^Re]­Re-MAE_3_-DOTA-FAPI demonstrating superior stability (80%) at 24 h compared to others (65–68%), confirming MAE_3_ as a more stable chelator for radiotracers. All four ^188^Re-FAPI tracers were hydrophilic (logD_7.4_: −1.78 to −2.97), with DOTA conjugation significantly enhancing hydrophilicity. Among them, [^188^Re]­Re-MAE_3_-DOTA-FAPI showed the most favorable hydrophilicity (logD_7.4_ = −2.97).

SPECT/CT imaging revealed distinct *in vivo* performance among the four ^188^Re-labeled FAPI tracers (Figure 2). At 1 h p.i., both [^188^Re]­Re-MAS_3_-FAPI and [^188^Re]­Re-MAE_3_-FAPI demonstrated predominant hepatobiliary clearance, with intense radioactivity accumulation in the liver, gallbladder, and intestines, along with notable thyroid uptake. This biodistribution pattern correlated with their relatively higher lipophilicity (logD_7.4_ = −1.78 and −2.71, respectively), which facilitated nonspecific uptake in these organs and resulted in suboptimal tumor targeting efficiency. The high thyroid uptake may be attributed to the release of free ^188^Re, suggesting limited *in vivo* stability for [^188^Re]­Re-MAS_3_-FAPI and [^188^Re]­Re-MAE_3_-FAPI. In contrast, [^188^Re]­Re-MAS_3_-DOTA-FAPI and [^188^Re]­Re-MAE_3_-DOTA-FAPI showed improved tumor uptake and retention. Among them, [^188^Re]­Re-MAE_3_-DOTA-FAPI exhibited the highest tumor uptake (tumor SUV_max_ of 0.84 ± 0.06 at 48 h p.i.), prolonged tumor retention, and lowest background signal. These findings revealed that the beneficial impact of DOTA conjugation, which enhances both tracers’ stability and hydrophilicity and improves pharmacokinetics and tumor accumulation. Strategic incorporation of DOTA, even in molecular constructs where it is not strictly essential for radionuclide chelation, can significantly optimize the in vivo performance of radiopharmaceuticals.^26,27^

**Figure 2:**
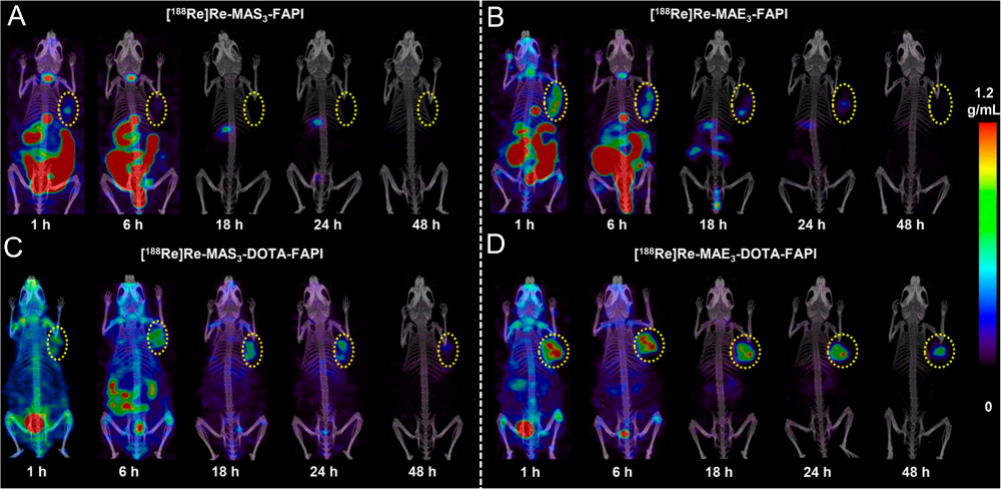
SPECT/CT imaging of [^188^Re]­Re-MAS_3_-FAPI (A), [^188^Re]­Re-MAE_3_-FAPI (B), [^188^Re]­Re-MAS_3_-DOTA-FAPI (C), and [^188^Re]­Re-MAE_3_-DOTA-FAPI (D) in HT1080-FAP tumor-bearing mice at 1, 6, 18, 24, and 48 h postinjection (p.i.). The figure was adapted from ref 25. Copyright 2025 American Chemical Society.

Surface plasmon resonance (SPR) analysis and competition binding assays were performed to evaluate the FAP affinity of four precursors. The *K*_D_ value of MAS_3_-FAPI, MAS_3_-DOTA-FAPI, MAE_3_-FAPI, and MAE_3_-DOTA-FAPI were 1.61 × 10^–12^, 3.52 × 10^–14^, 2.49 × 10^–11^, and 1.46 × 10^–10^ M, respectively. Their corresponding IC_50_ values of MAS_3_-FAPI, MAS_3_-DOTA-FAPI, MAE_3_-FAPI, and MAE_3_-DOTA-FAPI were 21.6 ± 2.34, 15.1 ± 1.17, 43.1 ± 3.75, and 24.2 ± 1.38 nM, respectively.^25^ The *in vivo* specificity of [^188^Re]­Re-MAS_3_-DOTA-FAPI and [^188^Re]­Re-MAE_3_-DOTA-FAPI was confirmed in HT1080-FAP (FAP-positive) and HT1080 (FAP-negative) tumor-bearing mice (Figure 3). At 3 h p.i., [^188^Re]­Re-MAS_3_-DOTA-FAPI and [^188^Re]­Re-MAE_3_-DOTA-FAPI showed markedly higher SUV_max_ values in HT1080-FAP tumors (0.96 ± 0.05 and 1.54 ± 0.08, respectively) than in FAP-negative HT1080 tumors (0.13 ± 0.17 and 0.55 ± 0.39), demonstrating their effective targeting of FAP-positive tumors. Compared with the more lipophilic [^188^Re]­Re-MAS_3_-DOTA-FAPI, which undergoes hepatobiliary excretion and shows partial ^188^Re dissociation, the highly hydrophilic [^188^Re]­Re-MAE_3_-DOTA-FAPI is predominantly metabolized via the kidneys with no detectable ^188^Re release, indicating superior *in vivo* stability. Biodistribution results also showed that [^188^Re]­Re-MAE_3_-DOTA-FAPI had higher tumor uptake and prolonged tumor retention. Therefore, as a ^188^Re chelator, MAE_3_ confers better pharmacokinetic properties and greater *in vivo* stability to the probe compared to MAS_3_.

**Figure 3:**
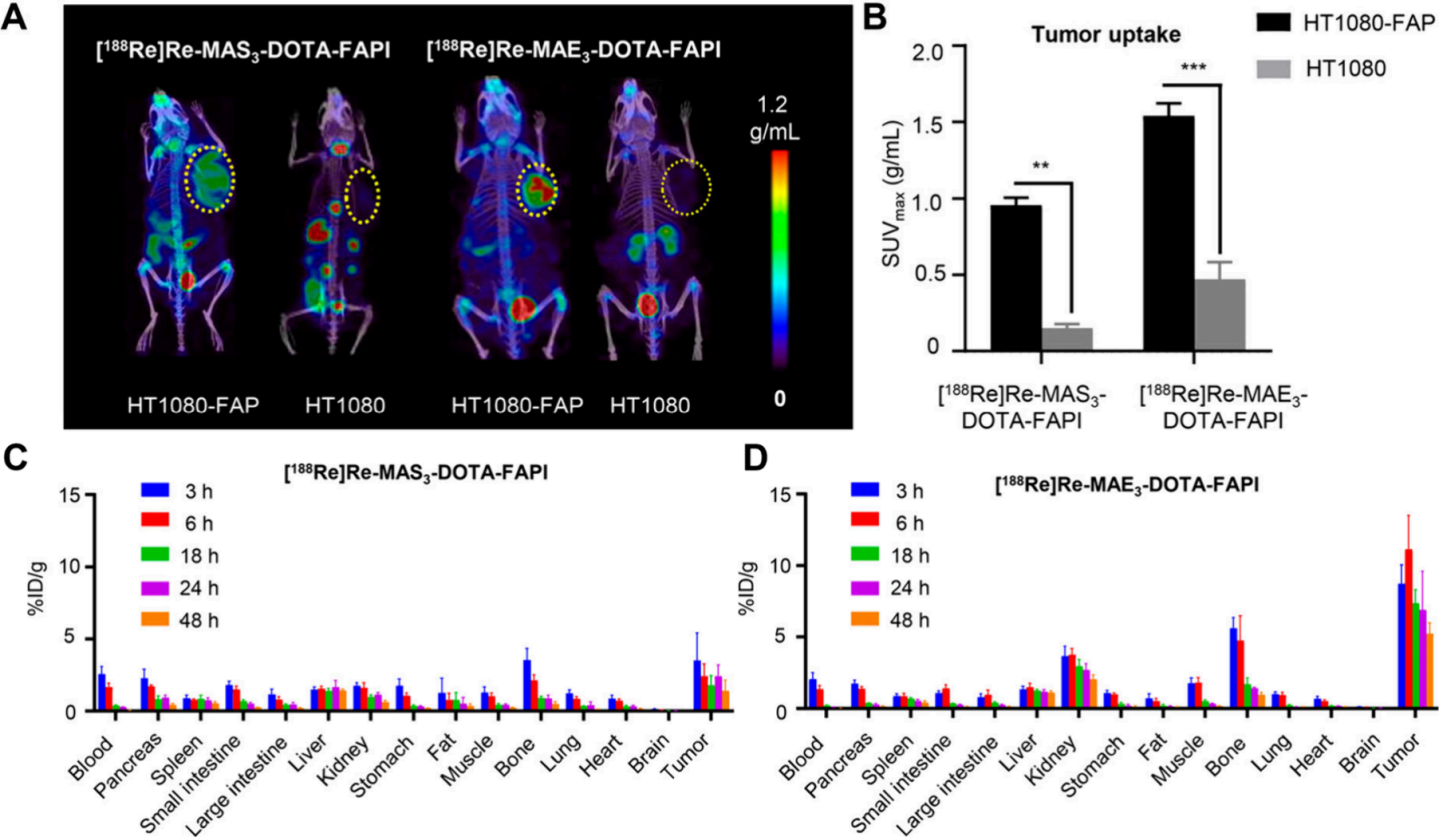
SPECT/CT imaging (A) and corresponding tumor uptake value (B) of [^188^Re]­Re-MAS_3_-DOTA-FAPI and [^188^Re]­Re-MAE_3_-DOTA-FAPI in HT1080-FAP and HT1080 tumor-bearing mice at 3 h p.i. Ex vivo biodistribution studies of [^188^Re]­Re-MAS_3_-DOTA-FAPI and [^188^Re]­Re-MAE_3_-DOTA-FAPI at 3, 6, 18, 24, and 48 h p.i. (C,D). The figure was adapted from ref 25. Copyright 2025 American Chemical Society.

Given the favorable pharmacokinetics of [^188^Re]­Re-MAE_3_-DOTA-FAPI, its therapeutic efficacy was evaluated in HT1080-FAP tumor-bearing mice using two dosing regimens (18.5 and 37 MBq) (Figure 4A). Compared to saline-treated controls, both dose groups showed rapid tumor growth suppression, with complete tumor regression observed in 3/7 mice (18.5 MBq) and 7/7 mice (37 MBq), and no recurrence through 80 days. No toxicity-related weight loss was observed in treated animals. Hematological toxicity was assessed 5 days after administration. Complete blood count (CBC) analysis at the higher dose of 37 MBq showed no significant differences in white blood cell (WBC) count, red blood cell (RBC) count, hemoglobin (HGB) level, or platelet (PLT) count when compared to controls, indicating the absence of treatment-induced myelosuppression (Figure 4B). Furthermore, histological examination via hematoxylin and eosin (H&E) staining of major organs revealed no noticeable histological abnormalities (Figure 4C). These findings, together with stable body weights, support the excellent safety profile for [^188^Re]­Re-MAE_3_-DOTA-FAPI.

**Figure 4:**
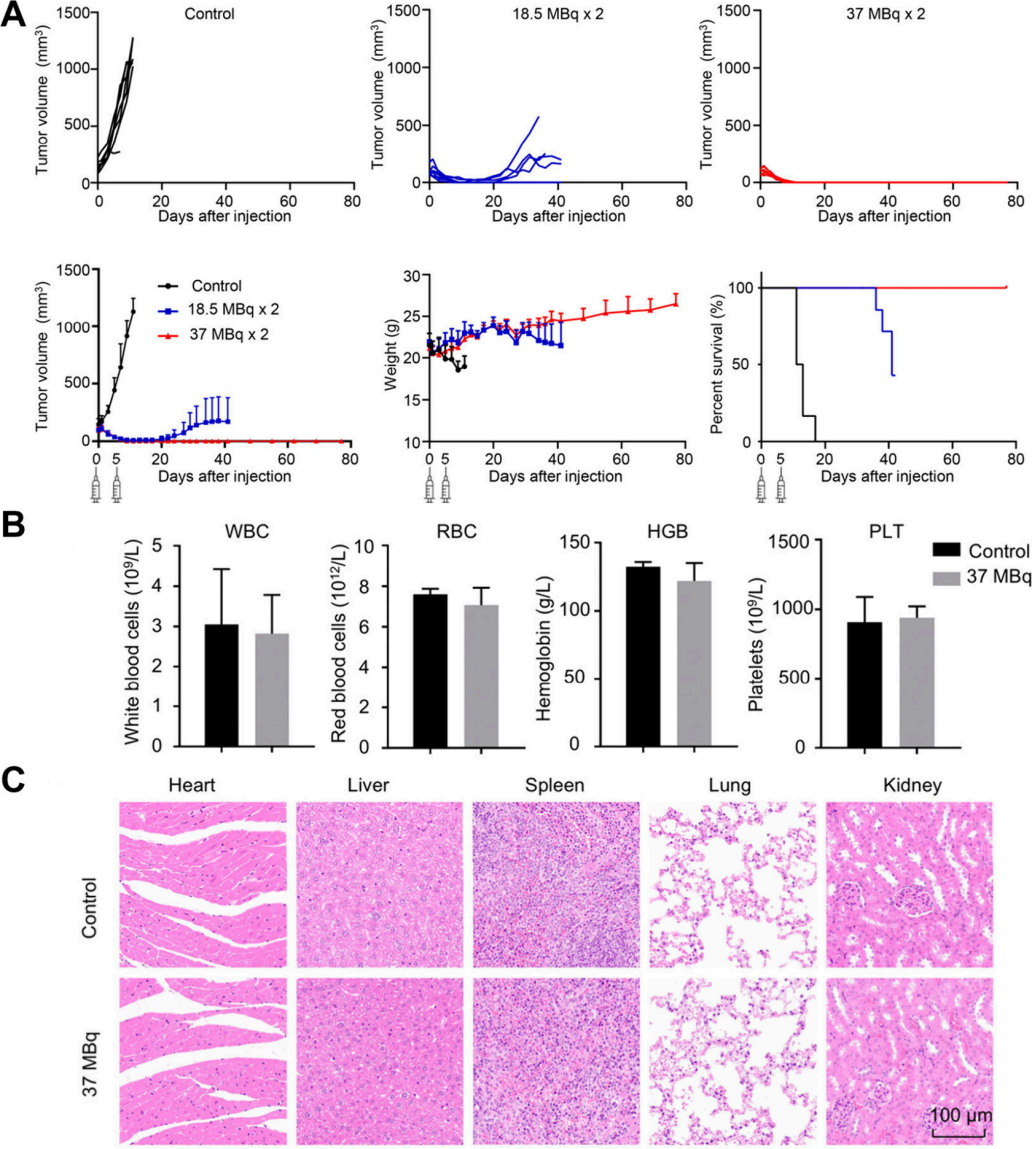
Radiotherapy study of [^188^Re]­Re-MAE_3_-DOTA-FAPI in HT1080-FAP tumor-bearing mice (A). Safety of Complete Blood Count (B) and H&E staining (C) for toxicity study. The figure was adapted from ref 25. Copyright 2025 American Chemical Society.

## Future Outlook

With its superior tumor targeting capability, optimal pharmacokinetics, and robust antitumor efficacy, [^188^Re]­Re-MAE_3_-DOTA-FAPI emerges as a highly promising candidate for FAP-TRT. However, technical considerations warrant attention: The current radiolabeling process using SnCl_2_ as the reducing agent may lead to formation of stable Sn-DOTA complexes, potentially compromising RCP.^28,29^ This highlights the need for developing alternative reduction strategies that maintain efficient perrhenate conversion while avoiding undesirable side reactions. Future direction should focus on three key areas: (1) Development of more robust ^188^Re-chelating systems to further enhance radiopharmaceutical stability; (2) Rational structural optimization of the FAPI scaffold to prolong intratumoral retention; and (3) Implementation of improved radiolabeling methodologies. These advancements will build upon the current success of ^188^Re-labeled FAPI tracers. Their cost-effectiveness and therapeutic efficacy position them to become attractive agents for oncology practice, particularly in low-resource settings where advanced treatment options remain inaccessible. Continued refinement of these targeted radiopharmaceuticals may establish them as promising therapies for FAP-expressing malignancies.

## Abbreviations

FAP: Fibroblast activation protein
CAF: cancer-associated fibroblasts
FAPI: FAP-inhibitor
TRT: targeted radionuclide therapy
SPECT: single photon emission computed tomography
DOTA: 1,4,7,10-tetraazacyclododecane-1,4,7,10-tetraacetic acid
MAG_3_: mercaptoacetyltriglycin
MAE_3_: mercaptoacetyl-Glu-Glu-Glu
MAS_3_: mercaptoacetyl-Ser-Ser-Ser
SPR: surface plasmon resonance
RCY: radiochemical yield
RCP: radiochemical purity
H&E: hematoxylin and eosin
WBC: white blood cell
RBC: red blood cell
HGB: hemoglobin
PLT: platelet
